# Development of a Temperate Climate-Adapted *indica* Multi-stress Tolerant Rice Variety by Pyramiding Quantitative Trait Loci

**DOI:** 10.1186/s12284-022-00568-2

**Published:** 2022-04-09

**Authors:** Na-Hyun Shin, Jae-Hyuk Han, Kieu Thi Xuan Vo, Jeonghwan Seo, Ian Paul Navea, Soo-Cheul Yoo, Jong-Seong Jeon, Joong Hyoun Chin

**Affiliations:** 1 Department of Integrative Biological Sciences and Industry, College of Life Sciences, Sejong University, Seoul, 05006 Korea; 2 Graduate School of Biotechnology and Crop Biotech Institute, Kyung Hee University, Yongin, Gyeonggi-do 17104 Korea; 3 Department of Plant Bioscience, College of Natural Resources and Life Science, Pusan National University, Miryang, 50463 Korea; 4 Plant Breeding, Genetics, and Biotechnology Division, International Rice Research Institute, Los Banos, Philippines; 5 Department of Plant Life and Environmental Science, Hankyong National University, Anseong, Gyeonggi-do 17579 Korea; 6 Life and Industry Convergence Research Institute, Pusan National University, Miryang, 50463 Korea

**Keywords:** Rice, *Pi9*, *Sub1*, *AG1*, Climate change, QTL pyramiding, Rice blast disease

## Abstract

Successful cultivation of rice (*Oryza sativa* L.) in many Asian countries requires submergence stress tolerance at the germination and early establishment stages. Two quantitative trait loci, *Sub1* (conferring submergence tolerance) and *AG1* (conferring anaerobic germination), were recently pyramided into a single genetic background, without compromising any desirable agronomic traits, leading to the development of Ciherang-Sub1 + AG1 (CSA). However, little research has been conducted to enhance plant tolerance to abiotic stress (submergence) and biotic stress (rice blast), which occur in a damp climate following flooding. The BC_2_F_5_ breeding line was phenotypically characterized using the AvrPi9 isolate. The biotic and abiotic stress tolerance of selected lines was tested under submergence stress and anaerobic germination conditions, and lines tolerant to each stress condition were identified through phenotypic and gene expression analyses. The Ciherang-Sub1 + AG1 + Pi9 (CSA-Pi9) line showed similar agronomic performance to its recurrent parent, CSA, but had significantly reduced chalkiness in field trials conducted in temperate regions. Unexpectedly, the CSA-Pi9 line also showed salinity tolerance. Thus, the breeding line newly developed in this study, CSA-Pi9, functioned under stress conditions, in which *Sub1*, *AG1*, and *Pi9* play a role and had superior grain quality traits compared to its recurrent parent in temperate regions. We speculate that CSA-Pi9 will enable the establishment of climate-resilient rice cropping systems, particularly in East Asia.

**Supplementary Information:**

The online version contains supplementary material available at 10.1186/s12284-022-00568-2.

## Background

Flooding is a frequent natural calamity in subtropical regions, and climate change can cause unexpected heavy rains and frequent inundation, which are expected to increase in Peninsular India and South Asia in the future (Mondal et al. 2020a, b). Rice (*Oryza sativa* L.) is the only plant species that can be grown in conditions prone to uncontrolled inundation or saline inundation; nonetheless, flooding is one of the major factors responsible for rice yield reduction (Hirabayashi et al. 2013; Kuanar et al. 2017). Rainfed lowland rice farms occupy 34–70% of the total rice production area in India and other South Asian countries, and more than 15 million ha of rainfed lowland rice systems are affected by flooding, decreasing rice productivity (Bailey-Serres et al. 2010; Emerick and Ronald 2019; Ali et al. 2006; Septiningsih et al. 2009). At least 16% of the rice yield is affected by flooding, accounting for a loss of more than one billion (USD) per year in South and Southeast Asia (Ram et al. 2002; Ali et al. 2006; Xu et al. 2006; Hirabayashi et al. 2013). Elevation in sea level, possibly due to global warming, has decreased rice cultivation near the coast, and the resultant frequent flooding has led to poor drainage, affecting the rice crop at all stages including germination and vegetative growth (Mackill et al. 2010). Most rice genotypes, including high-yielding varieties, cannot survive under deep standing water and cannot germinate under oxygen limiting conditions (Bailey-Serres et al. 2010; Baltazar et al. 2014). However, most modern varieties were developed without considering the need for germination and/or survival strategies under submerged conditions (Baltazar et al. 2019; Alam et al. 2020). A 34–83% yield reduction has been reported in modern varieties under stagnant flooding, but not fully submerged, conditions (Kato et al. 2019). In addition, compared to manual transplanting, direct seeding is becoming increasingly popular, as it is associated with reduced labor and operational costs, earlier flowering, shorter crop duration, and earlier maturity (by 7–10 days) (Baltazar et al. 2014; Kumar et al. 2015). However, one of the most serious problems in the open mechanized direct seeding system is early establishment, especially in wet seeding systems. Against those stresses, which are related with submergence in the germination and vegetative stages, two quantitative trait loci (QTLs) are required in modern *indica* rice varieties: *Submergence 1* (*Sub1*), which confers submergence tolerance by *Sub1A*; and *Anaerobic Germination-9-2* or *AG1*, which is associated with anaerobic germination and harbors the *TREHALOSE-6-PHOSPHATE PHOSHPHATASE 7* (*OsTPP7*) gene (Xu et al. 2006; Toledo et al. 2015; Alam et al. 2020). Among the genes identified at the *Sub1* QTL, *Sub1A* is found in a small collection of rice germplasm, whereas *Sub1B* and *Sub1C* are found in all rice varieties. *Sub1A* does not have any negative effects on rice yield and agronomic traits (Septiningsih and Mackill 2018). When *Sub1* was transferred to the mega rice variety Swarna, which is grown on approximately 5 million ha, the resulting genotype (Swarna-Sub1) produced a yield of 5–6 tons per ha in the absence of flooding, similar to Swarna (Emerick and Ronald 2019). Furthermore, 80–95% of Swarna-Sub1 plants survived and produced 45% higher yield than Swarna in areas flooded for 10 days (Emerick and Ronald 2019). Introgression of the *AG1* QTL, which improves germinative capacity under anaerobic conditions, is an effective approach for overcoming flooding-related problems at the germination stage including root establishment. IR64-AG1 near isogenic lines (NILs) showed 121% higher germination than IR64, irrespective of the flood duration (Lal et al. 2018). The *AG1* QTL has been introgressed into several rice varieties, such as the variety Dongan (Kim et al. 2019a, b), without causing any negative effects on yield under normal conditions (Toledo et al. 2015). Similarly, the *Sub1* QTL has been introgressed into various mega varieties including Samba Mahsuri, CR1009, Thadokkham, BR11, Bac Thom7, PSB Rc18, and M-202 (Septiningsih et al. 2009, 2013; Khanh et al. 2013; Xu et al. 2004). In addition, both *Sub1* and *AG1* QTLs have been pyramided together in Ciherang, IR64, Swarna, NSIC Rc222, and Bg358 (Toledo et al. 2015; Rafael et al. 2015; Sartaj et al. 2016), and combined with other abiotic stress tolerance QTLs/genes including *AG2*, *Pup1* (phosphorous uptake under rainfed/upland condition), *DTY* (drought), and *Saltol* (salinity) in the rice varieties IR64, Swarna, PSB Rc82, and Mahsuri (Mondal et al. 2020a, b; Shin et al. 2020; Singh et al 2016; Dar et al. 2014).

The rice variety Ciherang-Sub1 + AG1 (CSA), which was developed by the introgression of *Sub1* and *AG1* into Ciherang, was used in this study (Septiningsih et al. 2015; Toledo et al. 2015). Ciherang, developed at the International Rice Research Institute (IRRI; Philippines), is the predominant variety of Indonesia with a high yield potential (5–7 tons/ha) (IRRI 2015; Arief et al. 2018). The breeding line CSA, which is morphologically highly similar to Ciherang and contains *Sub1* and *AG1* for tolerance to submergence and anaerobic germination conditions, was selected using the marker-assisted backcrossing (MABC) approach, with five markers for the *Sub1* locus (RM8300, a simple sequence repeat [SSR] maker downstream of *Sub1A*, ART5, and three insertion/deletion [Indel] markers located within *Sub1C*) and four markers for the *AG1* locus (TPP_GE5, HPP400_410_3, Drebups6bp, and Drebdws4bp) (Toledo et al. 2015). CSA is tolerant to submergence at all growth stages and has nearly the same plant height and tiller number as Ciherang-Sub1 (Toledo et al. 2015). Based on genotype, CSA is approximately 99% similar to Ciherang, thus there is minimal interaction between the genetic backgrounds of Ciherang and the QTL donor (Toledo et al. 2015). However, compared with Ciherang, CSA exhibits delayed flowering (by approximately 6 days) and higher grain yield in one season (Toledo et al. 2015). This variation in flowering period of *Sub1*-introgressed lines was previously reported by Septiningsih et al. (2013) and demonstrates the limitation of marker-assisted selection (MAS), when used alone, in the selection of identical genotypes by introgressing useful traits into high-yielding varieties. Nonetheless, MAS provides the opportunity to explore new traits by testing multiple genetic combinations of QTLs with the endogenous genes of high-yielding or mega varieties.

Submergence tolerance, anaerobic germination and salinity tolerance are crucial value-adding abiotic stress-related traits. In coastal areas, nutrient-poor and saline soil conditions promote biotic stresses, such as rice blast, which challenge rice production by affecting plants after flowering. The high-level accumulation of abscisic acid (ABA) in plants grown in saline areas affects their biotic stress response and makes them susceptible to fungal pathogens, such as the rice blast pathogen *Magnaporthe oryzae* (Couch and Kohn. 2002; Ton et al. 2009; Jiang et al. 2010; Asano et al. 2012). *M. oryzae*, a filamentous ascomycete, is distinct from *Magnaporthe grisea*, and can infect rice plants at all growth stages by entering through leaves, stems, nodes, and panicles (Wilson and Talbot 2009). Rice blast, caused by *M. oryzae,* is the most devastating disease of rice, responsible for 10–35% of yield loss (Fisher et al. 2012). More than 100 blast resistance (*R*) genes have been mapped to date, and most *Pi* genes, which have been characterized as 25 *R* genes, encode nucleotide-binding site leucine-rich repeat (NBS-LRR) proteins, which are constitutively expressed in blast-resistant plants (Wilson and Talbot 2009; Li et al. 2019). Exceptions are *Pid2*, *Pi21* and *Ptr* which encode a receptor-like kinase protein, protein with heavy-metal-binding and proline-rich domains, and protein with four Armadillo repeats, respectively (Chen et al. 2006; Ballini et al. 2008; Zhao et al. 2018). Using *Pi* genes as part of a breeding process is slow and uneconomical because *M. oryzae* is a fast-evolving fungal pathogen that breaks the *R* gene-mediated resistance within 3–5 years (Miah et al. 2013; Li et al. 2019). Broad-spectrum resistance genes like *Pi9*, an *R* gene cloned from *O. minuta* J.Presl., a wild relative of cultivated rice, have significant potential to resist pests and diseases (Amante-Bordeos et al. 1992; Elgamal and Elshenawy 2018). The *Pi9* gene is more useful than other *Pi* genes because it shows horizontal resistance against a diverse range of blast races (Qu et al. 2006). Recently, Zhou et al. (2020) identified 13 novel *Pi9* alleles using 107 blast-resistant varieties. In addition, *Pi9* is generally more effective when combined with other *Pi* genes in various rice varieties, without trade-off effects like decreased yield and delayed flowering (Khanna et al. 2015; Xiao et al. 2017; Wu et al. 2019). This implies that pyramiding *Sub1*, *AG1* and *Pi9* in a single genetic background will facilitate rice cultivation via direct seeding in large coastal areas including those in Korea.

Korea harbors 1.2 million registered foreigners (Statistics Korea 2020), who prefer *indica* rice over *japonica* rice for consumption, thus promoting the diversification of rice cultivation. Ciherang is one of the most famous *indica* varieties, especially in Indonesia. Furthermore, the rise in temperature during the growing season may require Koreans to identify or develop varieties adaptable to subtropical conditions. Thus, the development of new varieties suitable for direct seeding in temperate regions is important, and pyramiding genes that induce tolerance to multiple biotic and abiotic stresses in rice is in high demand. The objective of this study was to introgress the broad-spectrum blast resistance gene, *Pi9*, into the flood tolerant *indica* variety, CSA and evaluate the newly developed germplasm, Ciherang-Sub1 + AG1 + Pi9 (CSA-Pi9) in the saline western coastal area of Korea.

## Results

### Biotic and Abiotic Stress-Resistance Pyramiding Scheme

**Fig. 1:**
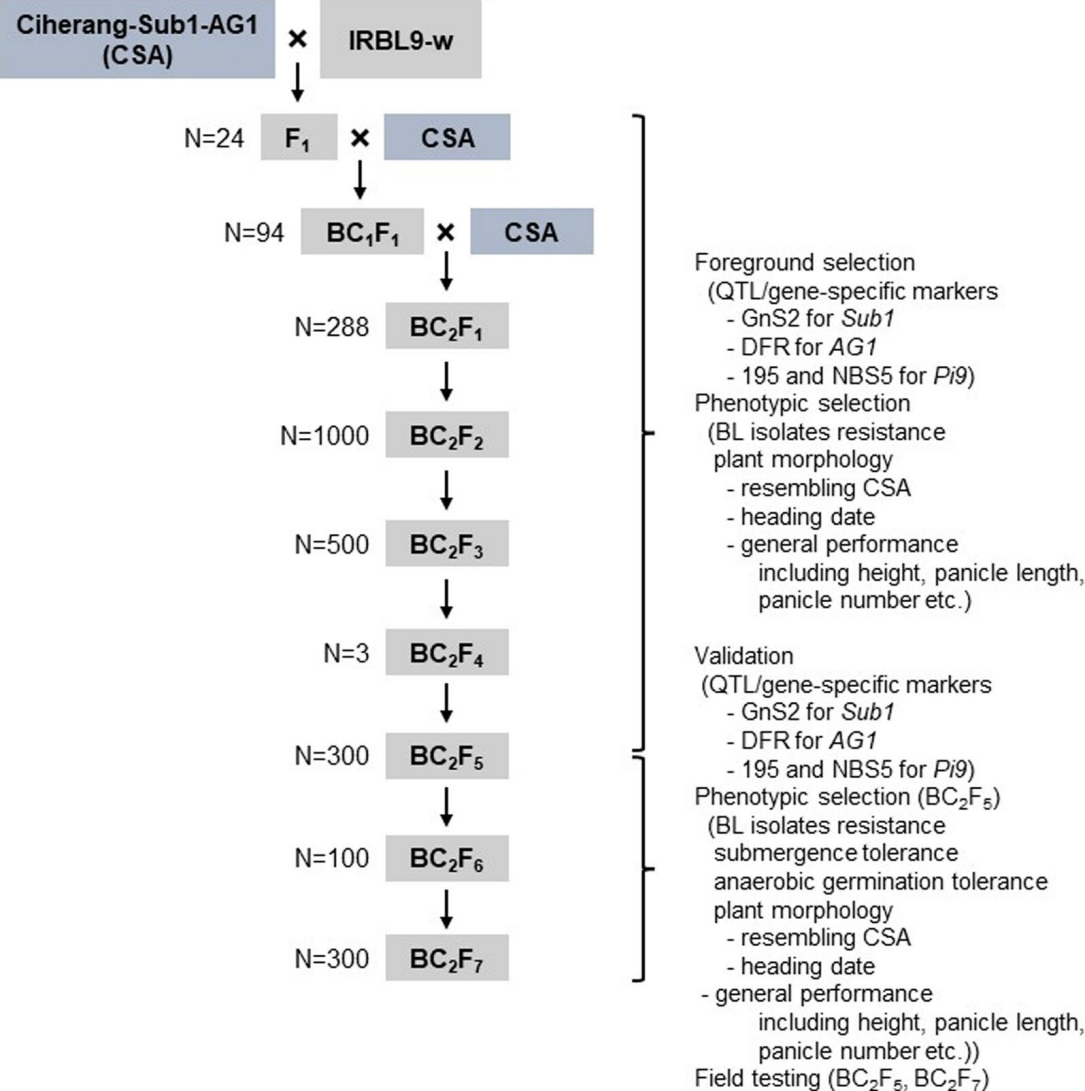
Schematic representation of the breeding strategy used to develop CSA-Pi9 via marker-assisted backcrossing (MABC). CSA: recurrent parent; IRBL9-w: donor parent; BL isolates: blast isolates

**Fig. 2:**
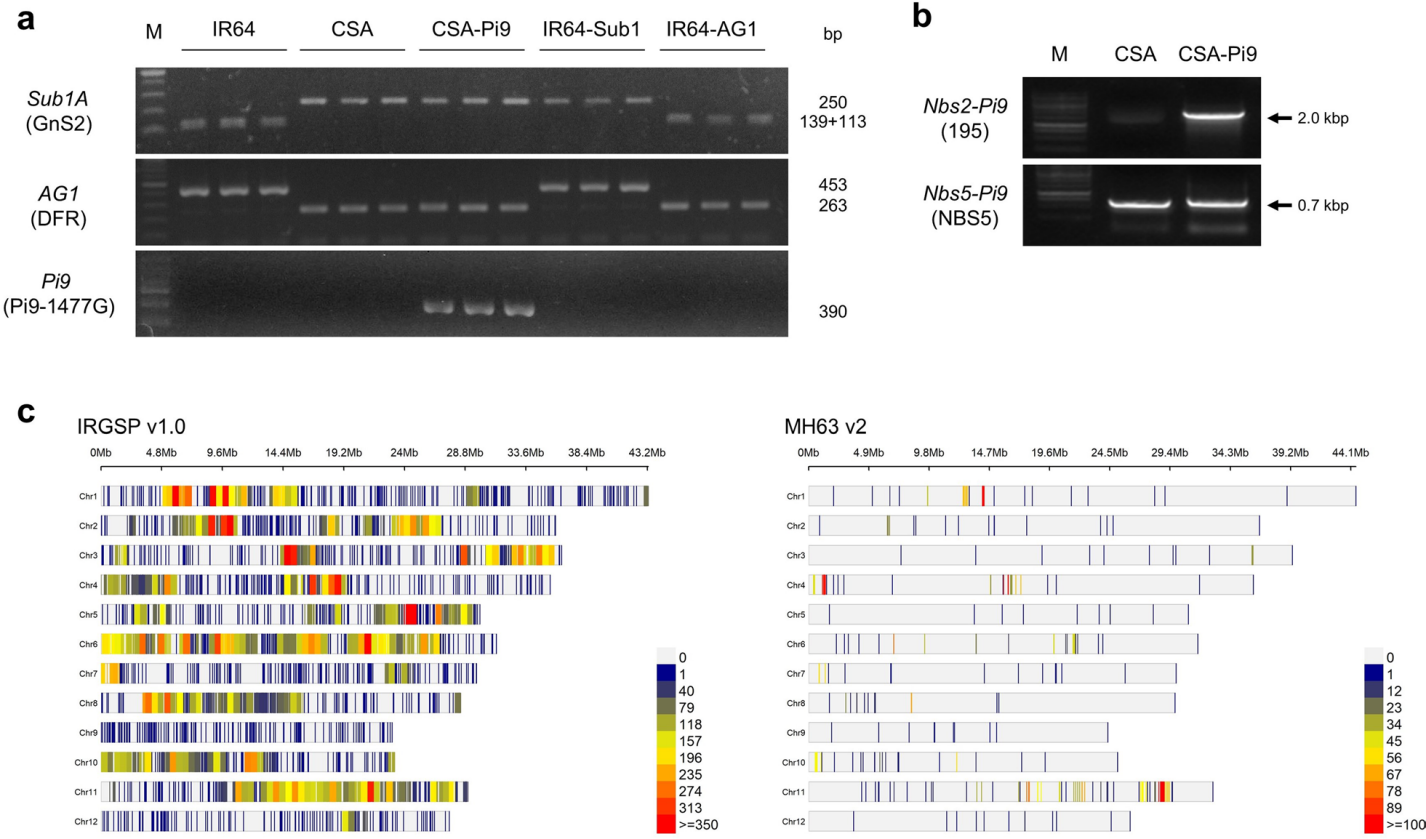
Analysis of the genetic background and foreground selection of CSA-Pi9. Foreground selection of CSA-Pi9 using *Sub1A-*, *AG1-* and *Pi9-*specific markers (**a**, **b**). Analysis of the genetic background of CSA-Pi9 using the KNU Axiom Oryza 580 K Genotyping Array containing SNPs designed on the basis of IRGSPv1.0 (left) and MH63 v.2 (right) (**c**). M: marker; bp: base pair; kbp: kilobase pair

IRBL9-w, a monogenic line has the *Pi9* resistance allele introgressed into the background of Lijiangxintuanheigu (LTH), a *japonica* variety which is extremely susceptible to blast, and CSA were crossed, and the resultant F_1_ individuals were backcrossed twice with CSA (Fig. 1). The backcross progeny that resembled the recurrent parent in plant type were selfed to generate the BC_2_F_7_ population. Screening for submergence tolerance and anaerobic germination was conducted in the BC_2_F_5_ generation. The genotype of BC_2_F_5_ and BC_2_F_7_ seeds was validated using QTL/gene-specific markers, and agronomic traits of BC_2_F_5_ and BC_2_F_7_ plants were tested under field conditions (Table 1). The BC_2_F_7_ plants were genotyped using GnS2 and DFR markers, which can specifically detect tolerance alleles of *Sub1* and *AG1* (Fig. 2a). Pi9-1477G and Nbs2-Pi9 (195), which are *Pi9*- and *Nbs2-Pi9*-specific markers, respectively, were used for the foreground genotyping of *Pi9* (Fig. 2a, b). The Pi9-1477G marker, which could distinguish between blast-resistant and susceptible varieties, was developed in this study by aligning *Pi9* alleles (Additional file 1: Fig. S1).

**Table 1:** Yield and yield-related traits of CSA-Pi9 and CSA plants grown under various paddy field conditions

Field location^1^	Variety	Generation	Growth period (DAT)^2^	Flowering period (DAT)^2^	Culm length (cm)^3^	Panicle length (cm)^3^	Panicle number^3^	Spikelet number^3^	Fertility (%)^3^	Hundred-grain weight (g)^3^	Grain yield^4^
Field A (2018)	CSA	–	146	64	90.67 ± 0.67	27.50 ± 0.50	9.67 ± 0.33	145.78 ± 6.41	89.03 ± 0.93	2.56 ± 0.03	25.49 ± 2.03
	CSA-Pi9	BC_2_F_5_	146	64	81.00 ± 1.73^*^	27.17 ± 0.60	11.00 ± 1.53	187.22 ± 9.02^**^	90.15 ± 1.34^***^	1.99 ± 0.01^***^	36.40 ± 0.53^*^
Field A (2019)	CSA	–	135	91	75.45 ± 0.57	25.02 ± 0.50	9.72 ± 0.47	141.56 ± 3.35	95.40 ± 0.76	2.66 ± 0.01	30.75 ± 1.22
	CSA-Pi9	BC_2_F_7_	135	76	66.83 ± 1.20^***^	25.40 ± 0.37	8.50 ± 0.47	182.67 ± 2.71^***^	93.64 ± 0.55	2.58 ± 0.01^**^	27.95 ± 1.43
Field B	CSA	–	128	92	73.40 ± 1.21	23.67 ± 1.20	15.33 ± 0.33	129.13 ± 3.11	65.95 ± 1.80	2.34 ± 0.02	32.63 ± 3.70
	CSA-Pi9	BC_2_F_7_	128	77	69.57 ± 1.07^**^	22.83 ± 0.48	14.67 ± 0.88	122.75 ± 5.39	93.29 ± 1.73^***^	2.19 ± 0.03^*^	32.00 ± 3.43
Field C	CSA	–	n.a.	n.a.	n.a.	n.a.	n.a.	n.a.	n.a.	n.a.	n.a.
	CSA-Pi9	BC_2_F_7_	141	91	n.d.	n.d.	n.d.	n.d.	n.d.	1.86 ± 0.04	18.56

^1^Field A: normal irrigation condition; Field B: normal irrigation condition in reclaimed region; Field C: high salinity condition in reclaimed region. Field locations are described in the “Materials and Methods” section

^2^Data represent the mean of five measurements. DAT: days after transplanting; n.a.: not applicable (all plants dead)

^3^Data represent mean ± standard error (SE; *n* = 5). Asterisks indicate statistically significant differences between CSA and CSA-Pi9 in each field (*P < 0.05, **P < 0.01, ***P < 0.001; Student's *t*-test). n.a.: not applicable (all plants died); n.d.: no data

^4^All values in this column represent grain yield per plant, estimated in grams (mean ± SE; *n* = 5; *P < 0.05; Student's *t*-test), except CSA-Pi9 in field C, which was harvested in bulk and total grain weight (334 g) was divided into the number of bulked plant (18 plants). This is the reason that there is no standard error in value of CSA-Pi9 in Field C

The genetic background of CSA-Pi9 was analyzed using the KNU Axiom Oryza 580 K Genotyping Array, which consists of single nucleotide polymorphisms (SNPs) designed on the basis of IRGSP v1.0. and MH63 v2 (Fig. 2c). CSA-Pi9 showed 99.2% and 89.6% CSA-specific alleles, based on IRGSP v1.0 and MH63 v2, respectively. Compared with other chromosomes, the ratio of CSA-specific alleles was lower on chromosome 6, which harbors *Pi9* (Additional file 3: Table S2).

### Resistance of CSA-Pi9 to Rice Blast

**Fig. 3:**
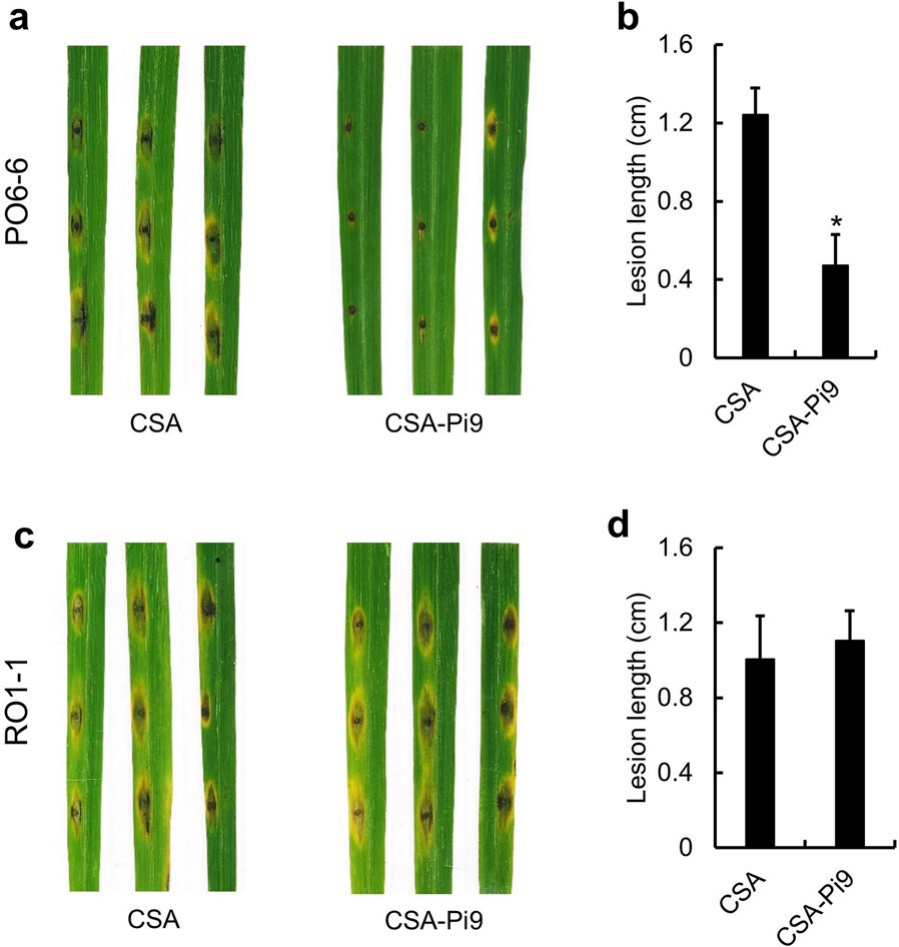
AvrPi9 phenotyping and validation of *Pi9*-mediated resistance. Photographs of lesions (**a**) and quantification of lesion length (**b**) in CSA-Pi9 and CSA leaves inoculated with PO6-6 (incompatible isolate). Photographs of lesions (**c**) and quantification of lesion length (**d**) in CSA-Pi9 and CSA leaves inoculated with RO1-1 (compatible isolate)

To examine blast resistance induced by *Pi9* derived from IRBL9-w, leaves of CSA-Pi9 and CSA (recurrent parent) were spot-inoculated with two rice blast isolates, PO6-6 (carrying the cognate effector of *Pi9*, Avrpi9; incompatible isolate) and RO1-1 (lacking AvrPi9; compatible isolate), as described previously (Kanzaki et al. 2002), and the size of lesions caused by the two isolates was compared at 9 days post-inoculation (dpi). When inoculated with PO6-6, lesions formed on CSA-Pi9 leaves were significantly smaller than those formed on CSA leaves (Fig. 3a, b), indicating that the introduced *Pi9* gene was effective in provoking effector-triggered immunity. However, CSA-Pi9 and CSA leaves inoculated with RO1-1 showed no significant difference in lesion size (Fig. 3c, d). Taken together, these data suggest that the introgression of *Pi9* into CSA induces resistance against AvrPi9-carrying isolates.

### Independent Functions of *Sub1* and *AG1* in CSA-Pi9 Under Each Stress Condition

**Fig. 4:**
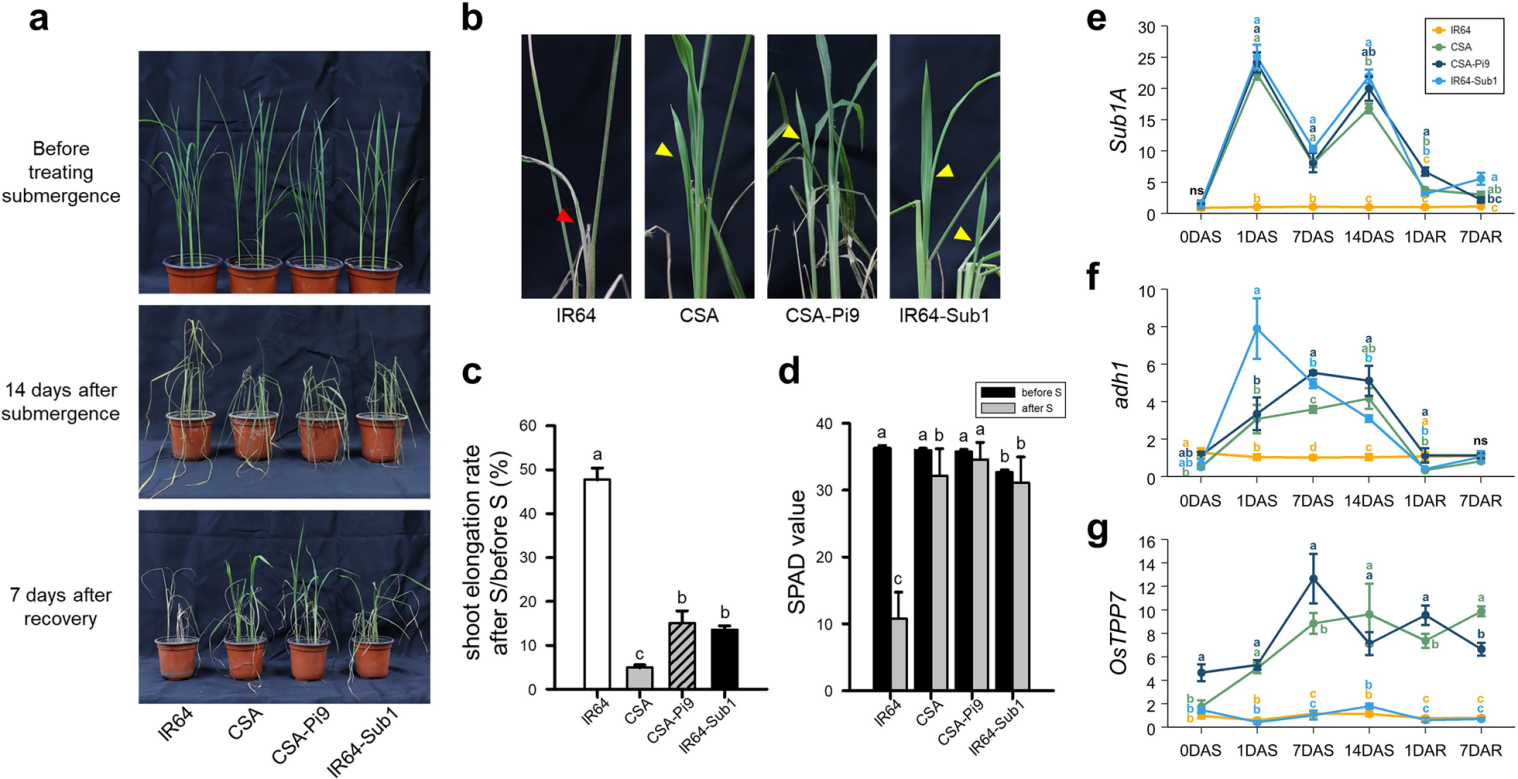
Characterization of the CSA-Pi9 breeding line treated with submergence stress for 14 days. Growth phenotype of CSA-Pi9 and other genotypes during submergence stress (**a**) and at 7 days after de-submergence (**b**). Shoot elongation rate [calculated as (shoot height after submergence (S)/shoot height before S) × 100] (**c**) and SPAD value (**d**) of CSA-Pi9 before and after submergence. Expression levels of *Sub1A* (**e**), *adh1* (**f**) and *OsTPP7* (**g**) under submergence stress. Data represent mean ± standard error (SE). Lowercase letters above bars and lines indicate statistically significant differences (*P* < 0.01; Duncan's multiple range test). IR64: submergence susceptible variety; IR64-Sub1: submergence tolerant variety; yellow arrow: newly emerging leaves; red arrow: no new leaves; DAS: days after submergence; DAR: days after recovery

To assess the submergence tolerance of CSA-Pi9, 2-week-old plants of CSA-Pi9, CSA, IR64, and IR64-Sub1 were submerged in 70-cm deep tap water for 14 days and subsequently recovered in shade for 7 days (Fig. 4a–d). CSA-Pi9, CSA, and IR64-Sub1, which harbor *Sub1*, recovered from submergence stress and sprouted new leaves at 7 days after recovery (DAR) (Fig. 4a, b). However, IR64, the susceptible control, dried after de-submergence (Fig. 4a, b). To evaluate whether *Sub1* restricts shoot elongation under submergence conditions (Xu et al. 2006), we measured the shoot length of plants before submergence and at 14 days after submergence (DAS) (Fig. 4c). CSA-Pi9 showed a submergence tolerant phenotype, with significantly reduced shoot elongation rate (18.6%), similar to IR64-Sub1 (13.6%). Shoot elongation rate was also reduced in CSA by 5.4%. However, IR64 showed the highest shoot elongation rate (47.7%). To determine the recovery of chlorophyll content, we measured the SPAD values of all genotypes pre- and post-submergence (Fig. 4d). The SPAD value of CSA-Pi9 was 34.6 after submergence and recovered to 97.4% of pre-submergence levels, which was higher than that observed in IR64-Sub1 (93.9%) and CSA (88.1%). Next, to determine the function of *Sub1* and *AG1* in CSA-Pi9, we examined the transcript levels of *Sub1A* and *OsTPP7*, the major genes underlying *Sub1* and *AG1* QTLs, respectively, and of *alcohol dehydrogenase1* (*adh1*) (Fig. 4e–g). Expression levels of *Sub1A* in CSA-Pi9 and IR64-Sub1 were similar until 7 DAS. At 14 DAS, although the expression level of *Sub1A* in CSA-Pi9 decreased slightly compared with IR64-Sub1, it was still higher than that in CSA (Fig. 4e). *Sub1A* was expressed until 7 DAR in *Sub1* lines (Fig. 4e). The expression level of *adh1* in IR64-Sub1 was significantly higher than that in CSA-Pi9 and CSA at 1 DAS but decreased gradually until 1 DAR (Fig. 4f). The expression of *adh1* was not strong in CSA-Pi9 at 1 DAS; however, it increased at 7 DAS and lasted until 14 DAS (Fig. 4f). After recovery, *adh1* expression decreased, and showed no significant difference among the various genotypes. The *OsTPP7* gene was expressed in CSA-Pi9 and CSA, and its expression level in CSA-Pi9 was similar to, or slightly higher than that observed in CSA under submergence conditions (Fig. 4g).

**Fig. 5:**
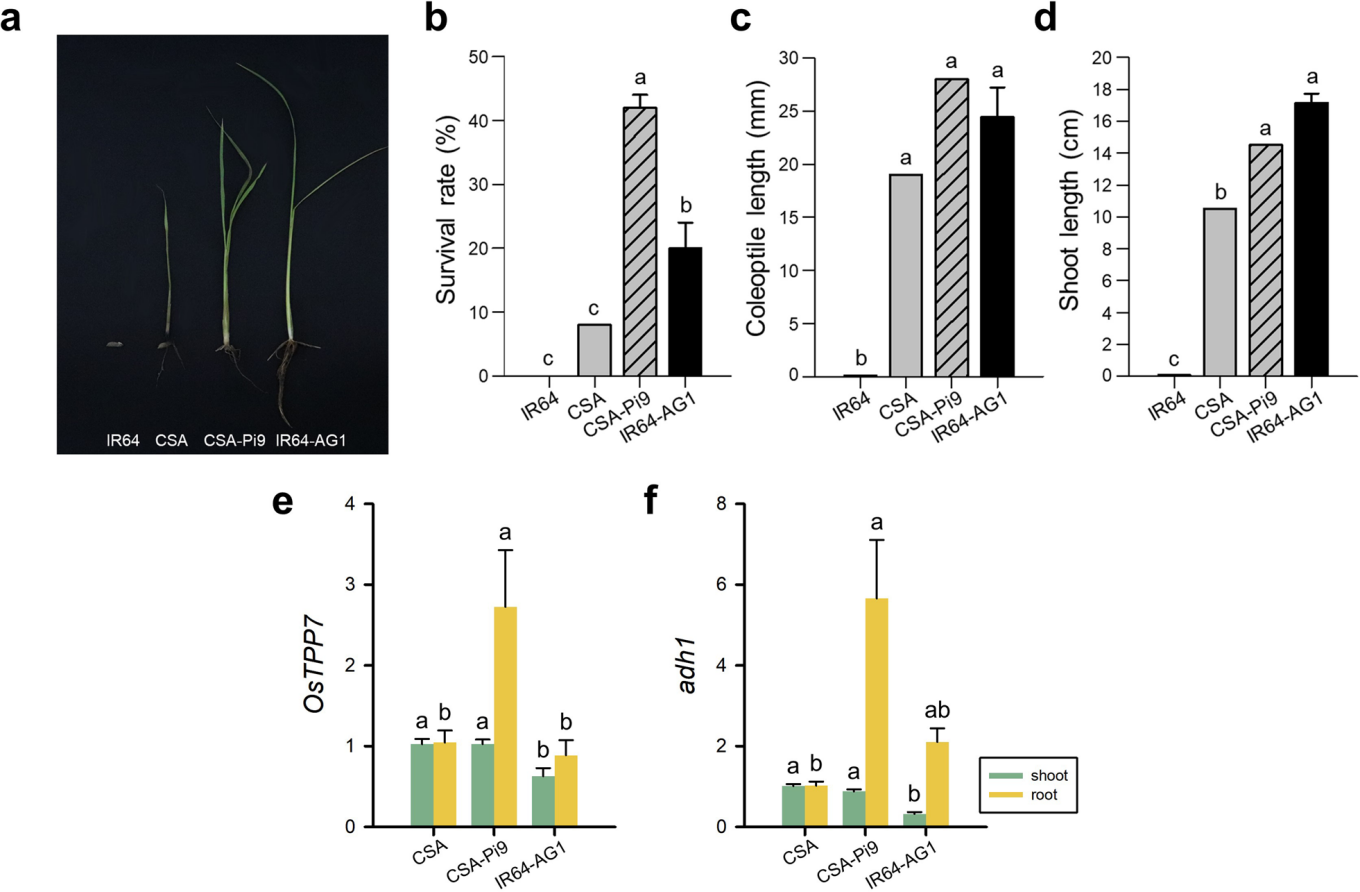
Characterization of CSA-Pi9 under anaerobic germination conditions. Phenotype of CSA-Pi9 plants at 34 days after sowing (**a**), germination rate (**b**), coleoptile length (**c**) and shoot length (**d**). Expression levels of *OsTPP7* (**e**) and *adh1* (**f**). Data for IR64 were unavailable, as its seeds did not germinate. Lowercase letters above bars indicate statistically significant differences (*P* < 0.01; Duncan's multiple range test)

To assess the function of *AG1* in CSA-Pi9, the germination of CSA-Pi9, CSA, IR64, and IR64-AG1 seeds was tested under anaerobic conditions (Fig. 5). While CSA-Pi9, CSA, and IR64-AG1 seeds germinated under anaerobic conditions, none of the IR64 seeds germinated (Fig. 5a), as expected. Additionally, CSA-Pi9 seedlings showed a significantly higher survival rate (42%) than CSA (8%) and IR64-AG1 (20%) seedlings (Fig. 5b). Significant differences were neither observed in coleoptile length among the germinated lines (~ 28 mm) nor in shoot length between CSA-Pi9 (14.5 cm) and IR64-AG1 (17.1 cm) (Fig. 5c, d). We also analyzed the expression of *OsTPP7* and *adh1* in the root and shoot separately. The shoot of CSA and CSA-Pi9 expressed *OsTPP7* and *adh1* to higher levels than that of IR64-AG1, while CSA-Pi9 root expressed both genes to higher levels than CSA and IR64-AG1 roots (Fig. 5e, f). Thus, CSA-Pi9 recovered fully from submergence stress and sprouted new leaves, and also showed powerful germinability under anoxic conditions (Figs. 4, 5). Based on these results, we conclude that both *Sub1* and *AG1* are functional in CSA-Pi9 under each stress condition, regardless of the presence of *Pi9*.

### Increased Spikelet Number of CSA-Pi9 Helps Maintain Grain Yield in Paddy Fields

**Fig. 6:**
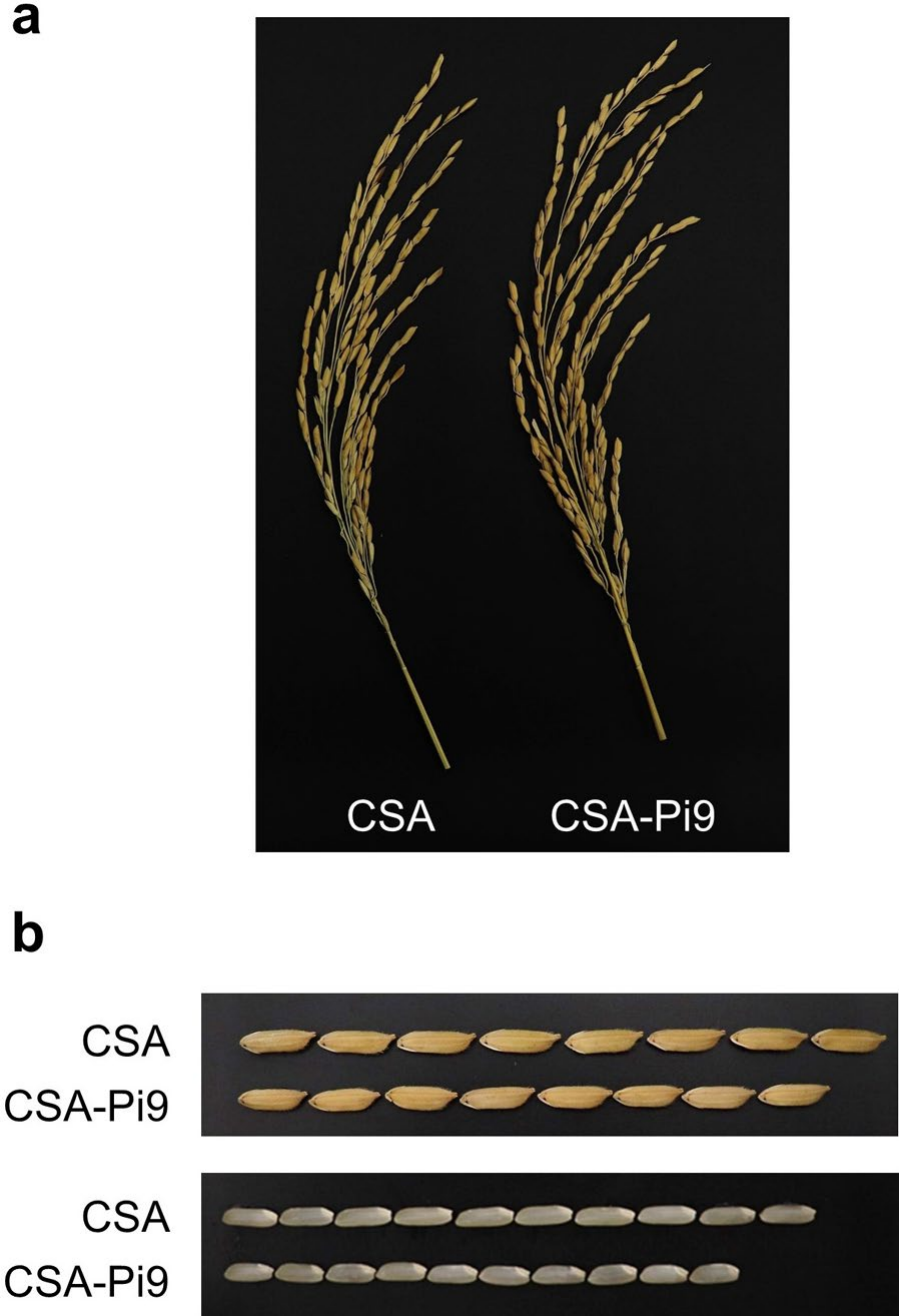
Comparison of the panicle shape (**a**) and grain shape with and without the hull (**b**) of CSA-Pi9 and CSA plants grown in field A (normal irrigation condition) in 2019

To investigate the agronomically important plant- and seed-related traits of CSA-Pi9 and CSA, plants of both genotypes were grown in paddy fields located in three different regions: field A (Suwon, normal growth conditions), field B (Seosan, normal growth conditions), and field C (Seosan, saline conditions). Plants in field A were tested for 2 years (2018 and 2019), whereas those in fields B and C were evaluated only for 1 year (2019). Plants in field A were grown for 146 days in 2018 and for 135 days in 2019. In field A, CSA-Pi9 and CSA plants flowered at the same date in 2018 (i.e., at 64 days after transplanting [DAT]); however, CSA-Pi9 flowered 15 days earlier than CSA plants in 2019 (76 and 91 DAT, respectively). In fields B and C, plants of both genotypes were grown for 135 and 128 DAT, respectively. In field B, CSA-Pi9 plants flowered 15 days earlier than CSA plants (77 and 92 DAT, respectively); however, in field C, CSA-Pi9 plants flowered at 91 DAT. No significant differences were observed between CSA-Pi9 and CSA in the length, number and architecture of panicles; however, the culm length of CSA-Pi9 plants was significantly shorter than that of CSA plants (Table 1, Fig. 6a).

In field A, CSA-Pi9 plants produced 187.22 and 182.67 spikelets per panicle in 2018 and 2019, respectively, which were 28.4% and 29.0% more, respectively, than those produced by CSA; however, in field B, the two genotypes showed no significant difference in spikelet number per panicle (Table 1). The fertility of CSA-Pi9 was the same or higher than that of CSA in fields A and B (Table 1). Compared with CSA, the hundred-grain weight of CSA-Pi9 was 22.3% lower in field A in 2018, and 3.0% and 6.4% lower in fields A and B, respectively, in 2019 (Table 1). However, the grain yield per plant of CSA-Pi9 was comparable with that of CSA in every field test, except in field A in 2018, where it was higher than that of CSA (Table 1). These results confirmed that the yield of CSA-Pi9 was comparable or preferable to CSA considering spikelet number per panicle and fertility, even though its hundred-grain weight was significantly lower than that of CSA. (Table 1).

In field C, which was located in a reclaimed saline region, CSA-Pi9 showed good yield performance, whereas none of the CSA plants survived. The flowering period of CSA-Pi9 in field C was 91 DAT, which was 14 days later than that in field B. The hundred-grain weight and grain yield of CSA-Pi9 was 1.86 g and 18.56 g per plant, respectively (Table 1). The tolerance of CSA-Pi9 to salinity stress was an unexpected finding.

### Superior Quality of CSA-Pi9 Grains with Low Chalkiness

Next, we investigated the grain quality traits of CSA-Pi9 and CSA, including grain size, chalkiness, amylose content, alkali spreading value and protein content (Table 2). The width of CSA-Pi9 grains was similar to the CSA grains, whereas the length of CSA-Pi9 grains was 9.3% and 13.1% shorter than the CSA grains in fields A and B, respectively, thus the length to width ratio was lower in CSA-Pi9 (Table 2, Fig. 6b). In fields A and B, CSA-Pi9 showed brown rice recovery rates of 76.7% and 78.5%, respectively, which were 3.5% and 5.0% lower than those of CSA, respectively (Table 2). Chalkiness is a major index of grain quality. CSA-Pi9 grains showed 33.6% and 38.6% less chalkiness than CSA grains in fields A and B, respectively (Table 2). The amylose content and alkali spreading value of CSA-Pi9 grains were lower than those of CSA grains in fields A and B, and significant differences in amylose content and alkali spreading value were observed, respectively, between fields A and B for both genotypes (Table 2). No significant difference in protein content was detected between CSA-Pi9 and CSA in fields A and B (Table 2).

**Table 2:** Grain quality traits of CSA-Pi9 and CSA plants grown in 2019 under different field conditions

Field location^1^	Variety	Generation	Grain length (cm)^2^	Grain width (cm)^2^	Length/width ratio^2^	Brown rice recovery (%)^2^	Chalkiness (%)^2^	Amylose content (%)^2^	Alkali spreading value^3^	Protein content (%)^2^
Field A	CSA	–	9.53 ± 0.06	2.46 ± 0.02	3.87 ± 0.03	79.53 ± 0.18	26.29 ± 2.58	29.44 ± 0.08	2.5	6.15 ± 0.28
	CSA-Pi9	BC_2_F_7_	8.64 ± 0.13***	2.45 ± 0.04	3.53 ± 0.04***	76.73 ± 0.24**	17.45 ± 2.44*	27.85 ± 0.15**	2.3	6.67 ± 0.27
Field B	CSA	–	9.81 ± 0.06	2.42 ± 0.02	4.06 ± 0.05	82.67 ± 0.57	32.59 ± 2.76	29.08 ± 0.67	4	8.87 ± 0.28
	CSA-Pi9	BC_2_F_7_	8.52 ± 0.07***	2.35 ± 0.03	3.63 ± 0.04***	78.53 ± 0.13*	20.00 ± 3.79*	27.86 ± 0.07	2***	7.82 ± 0.33
Field C	CSA	–	n.a.	n.a.	n.a.	n.a.	n.a.	n.a.	n.a.	n.a.
	CSA-Pi9	BC_2_F_7_	8.27 ± 0.08*	2.34 ± 0.03	3.54 ± 0.04	77.6 ± 0.23*	26.03 ± 3.34	23.15 ± 0.17***	4***	13.06 ± 0.17***

^1^Field A: normal irrigation condition; Field B: normal irrigation condition in reclaimed region; Field C: high salinity condition in reclaimed region. Field locations are described in the Materials and Methods

^2^Data represent mean ± SE (*n* = 5). Asterisks indicate statistically significant differences between CSA and CSA-Pi9 in each field (*P < 0.05, **P < 0.01, ***P < 0.001; Student's *t*-test). n.a.: not applicable (all plants dead)

^3^Data represent the mean of five measurements. n.a.: not applicable (all plants died)

To evaluate the effect of salinity on grain quality in rice, we compared the grain quality of CSA-Pi9 between fields B and C (Table 2). The grain length and brown rice recovery rate of CSA-Pi9 in field C were slightly lower than those in field B (Table 2). The chalkiness of CSA-Pi9 grains showed no significant difference between the two fields. Compared with field B, the amylose content of CSA-Pi9 grains produced in field C was lower by 16.9%, whereas the alkali spreading value and protein content were significantly higher (100% and 67.0%, respectively) (Table 2). These data suggest that CSA-Pi9 exhibits superior grain quality traits compared with CSA, with decreased chalkiness under both normal and saline conditions, even though the grain size of CSA-Pi9 was smaller than that of CSA because of shortened grain length.

## Discussion

### *Indica* Rice and Its Availability in Temperate Regions

Owing to global warming, the climate of regions such as Korea is changing from temperate to subtropical; however, very few studies have evaluated rice germplasm as single plants across temperate and tropical regions (Chung et al. 2004; Ha et al. 2011; Huang et al. 2021; Navea et al. 2017; Takai et al. 2019). In addition, most of the stress tolerance QTLs/genes, such as *Sub1*, *AG1*, and *Pi9*, have been derived from rice germplasm adapted to tropical regions, and are rarely found in modern rice varieties (Ismail et al. 2013; Baltazar et al. 2019; Ning et al. 2020). Evaluation of rice plants adapted to tropical regions is difficult in temperate regions, because of variation in climate-sensitive traits such as plant type and flowering time (Ha et al. 2011). However, the *indica* rice accession developed in this study, CSA-Pi9, was well adapted to temperate regions. The introgressed *Sub1*, *AG1*, and *Pi9* loci of CSA-Pi9 functioned normally under temperate climate conditions, without compromising any agronomic trait, and the tolerance of CSA-Pi9 to anaerobic and submergence conditions was similar to that of CSA under tropical climate conditions (Figs. 4, 5; Toledo et al. 2015). Previously, CSA showed a survival rate of approximately 47.3% under anaerobic conditions in a tropical region (Toledo et al. 2015); however, in this study conducted in temperate regions, the survival rate of CSA decreased to 8% (Fig. 5b). By contrast, CSA-Pi9 showed a survival rate of 42%, and expressed the *OsTPP7* gene in roots germinated under anaerobic conditions in temperate regions (Fig. 5b). In addition, compared with CSA, CSA-Pi9 seedlings exhibited full recovery after de-submergence with less chlorosis, and showed significantly higher expression levels of three genes (*Sub1A*, *OsTPP7*, and *adh1*) than those of CSA during submergence stress in the temperate region (Fig. 4). Additionally, we noticed that *Sub1* and *AG1* were functional in temperate climate, and the tolerance levels were similar to those of *Sub1* and *AG1* in tropical conditions.

### Advantages Acquired by a Molecular Breeding of CSA-Pi9

The development and utilization of adequate molecular markers for genotyping target loci and whole-genome background are essential for modern molecular breeding programs. However, the development of allele-specific markers for the *Pi9* gene is difficult, because it contains repeat sequences. In this study, the SNP marker Pi9_1477G was used to discriminate among various *Pi9* alleles during MABC. However, because of high sequence similarity between Nbs5 and Nbs2 at the primer-binding sites, Pi9-1477G amplified the Nbs5 region instead of Nbs2 in some steps of the selection process in the current study; for example, BC_2_F_2_-generation plants harboring the *Nbs5-Pi9* allele were selected with Pi9_1477G (data not shown). Therefore, the application of multiple gene-specific markers is recommended to identify the *Nbs2-Pi9* allele.

Unpredictable climate change imposes multiple biotic/abiotic stresses on plants, and most elite varieties are susceptible to these stresses. However, owing to the development of markers associated with several important traits, it is possible to pyramid multiple traits into one cultivar using the MABC approach (Das and Rao 2015; Das et al. 2018; Dixit et al. 2020). Several attempts have been made to develop multiple biotic/abiotic-stress resistant elite varieties (Chukwu et al. 2019; Chithrameenal et al. 2018; Feng et al. 2018; Sandhu et al. 2020). Nevertheless, only a few varieties have been developed that exhibit multi-stress resistance along with superior quality traits in the background of Tapaswini, Swarna and White Ponni as summarized in Table 3 (Das et al. 2018; Dixit et al. 2020; Muthu et al. 2020). Therefore, we introgressed *Pi9* into CSA using the MABC method to improve not only abiotic/biotic stress tolerance but also agronomically important traits like culm length and flowering period in addition to quality related traits. The CSA-Pi9 breeding line developed in this study showed tolerance to submergence and anaerobic germination conditions and to blast, as shown by phenotypic and gene expression analyses. The yield of CSA-Pi9 was similar to that of CSA under normal field conditions, even though CSA showed higher hundred grain weight compared with CSA-Pi9 (Table 1). Moreover, the chalkiness of CSA-Pi9 was significantly lower than that of CSA in all field tests (Table 2), which implies that the quality of CSA-Pi9 grains is superior to that of CSA grains. However, CSA-Pi9 and CSA showed no polymorphic SNPs in *Chalk5*, *GWC1*, and *FLR1*, the representative genes affecting grain chalkiness (Guo et al. 2020; Li et al. 2014; Pu et al. 2017). Recently, breeders were challenged to develop a high yielding, climate change-ready rice variety, with good eating quality and nutritional composition as cooked rice (Collard et al. 2019; Manangkil et al. 2020). The CSA-Pi9 breeding line generated in this study represents an important genetic material that could be used not only in future rice breeding programs, but also for commercial cultivation in Asian countries as a multi-stress resistant cultivar with enhanced grain quality and agronomic performance.

**Table 3:** Combination of multi-stress resistant QTLs/genes with superior quality traits

Line	Introgressed QTLs/genes	Pyramided stress tolerance	Total no. of pyramided QTLs/genes	Generation	Background	References
ITGP1	*Xa4, xa5, xa13, Xa21, Gm1, Gm4, Pi2, Pi9, Sub1, Saltol*	Bacterial blight, gall midge, blast, submergence, salinity	10	BC_3_F_3_	Tapaswini	Das et al. (2018)
ITGP2						
ITGP4						
ITGP5						
ITGP7	*Xa4, xa5, xa13, Xa21, Gm1, Gm4, Sub1, Saltol*	Bacterial blight, gall midge, submergence, salinity	8			
ITGP8	*Xa4, xa5, xa13, Xa21, Gm1, Gm4, Pi2, Sub1, Saltol*	Bacterial blight, gall midge, blast, submergence, salinity	9			
ITGP9	*Xa4, xa5, xa13, Xa21, Sub1, Saltol*	Bacterial blight, submergence, salinity	6			
ITGP10						
ITGP14						
ITGP20						
IL1	*Pi9, Xa4, xa5, Xa21, Bph17, Gm8, qDTY1.1, qDTY3.1*	Bacterial leaf blight, blast, brown planthopper, gall midge, drought	8	IC_3_F_7_	NIL-Swarna + drought	Dixit et al. (2020)
IL2	*Pi9, Xa4, xa5, Bph3, Gm4, Gm8, qDTY1.1, qDTY3.1*					
IL3	*Pi9, Xa4, xa5, Xa21, Bph3, Bph17, Gm4, qDTY1.1, qDTY3.1*	Bacterial leaf blight, blast, brown planthopper, gall midge, drought	9			
IL4	*Xa4, xa5, Bph3, Bph17, Gm4, qDTY1.1, qDTY3.1*	Bacterial leaf blight, brown planthopper, gall midge, drought	7			
IL5	*Xa4, xa5, Xa21, Bph3, Gm4, qDTY1.1, qDTY3.1*					
IL6	*Pi9, Xa4, xa5, Xa21, Bph3, Bph17, Gm4, Gm8, qDTY1.1, qDTY3.1*	Blast, Bacterial leaf blight, brown planthopper, gall midge, drought	10			
IL7	*Xa4, xa5, Xa21, Bph3, Gm4, qDTY1.1, qDTY3.1*	Bacterial leaf blight, brown planthopper, gall midge, drought	7			
BIL # 3-11-9-2	*qDTY1.1, qDTY1.2, qDTY1.3, qDTY2.1, qDTY3.1, qDTY6.1*	Drought	6	BC_1_F_4_	Improved White Ponni	Muthu et al. (2020)
BIL # 3-11-11-1	*qDTY1.1, qDTY2.1, Saltol, Sub1*	Drought, salinity, submergence	4			
BIL # 3-11-11-2						

### Salinity Tolerance of CSA-Pi9

Traits not observed in the parental genotypes are occasionally observed in the progeny. In the current study, the CSA-Pi9 line showed salinity tolerance, which was a serendipitous finding in field trials. The CSA-Pi9 line was well adapted to the reclaimed land at the seashore, where growth conditions were humid and saline (Tables 1, 2). Because CSA was not adapted to the reclaimed land, we could assume that the salt tolerance of CSA-Pi9, which was derived from the introgression of *Pi9* into CSA, was conferred by the *Pi9* gene. However, the salt tolerance of *O. minuta* (*Pi9* donor) and IRBL9-w has not been reported to date. To determine whether the salinity tolerance of CSA-Pi9 is derived from IRBL9-w, we examined the salinity tolerance of IRBL9-w using a hydroponic system, since IRBL9-w has not been tested in a reclaimed area. However, IRBL9-w was sensitive to salt concentrations of 0.6% and 1.0% (data not shown), suggesting that the salinity tolerance of CSA-Pi9 was not derived from IRBL9-w. Next, we compared the *Saltol* QTL region (10.8–16.4 Mb on chromosome 1) between CSA-Pi9 and CSA lines and identified 1192 SNPs (data not shown). Mansuri et al. (2020) reported candidate genes involved in salt tolerance, based on the integrative meta-analysis of RNA-seq and microarray data available at the National Center for Biotechnology Information (NCBI) database. Axiom Oryza 580 K Genotyping Array data was used to analyze the presence of polymorphic SNPs between CSA-Pi9 and CSA in gene listed in Mansuri et al. (2020). The results revealed 27 SNPs between CSA and CSA-Pi9 (Additional file 4: Table S3). One of these 27 SNPs was located in the pectinesterase gene, which belongs to the *Saltol* QTL region. However, because the *Saltol* QTL harbors many genes and has not been fully characterized, it is difficult to conclude which genes are the major contributors to salt tolerance in CSA-Pi9. The salinity tolerance of CSA-Pi9 could be caused by one or more of the candidate genes listed in Additional file 4: Table S3 or by the interactions among the introgressed QTL/genes. In the future, we plan to determine the mechanism of salinity resistance in CSA-Pi9. We speculate that the CSA-Pi9 line could be recommended to farmers who use the direct seeding method for cultivating rice in saline soil in East Asia under high humidity conditions, which promote rice blast.

## Conclusions

In this study, the breeding line CSA-Pi9, was developed by introducing *Pi9*, a broad-spectrum blast resistance gene, from *O. minuta* into CSA. CSA-Pi9 harbors 89.6% of the CSA (recurrent parent)-specific alleles. The *Sub1*, *AG1*, and *Pi9* QTLs/genes were expressed and functional in CSA-Pi9 under submergence stress and anaerobic germination conditions. The tolerance of CSA-Pi9 to salinity stress was a serendipitous finding. Compared with CSA, CSA-Pi9 showed similar yield, improved grain quality with less chalkiness when grown in a temperate region. Thus, the CSA-Pi9 breeding line developed in this study could be used as a useful donor in breeding programs aimed at developing multi-stress resistant varieties for cultivation in temperate regions.

## Materials and Methods

### Plant Materials

CSA (GID 4537744) harbors *Sub1* and *AG1* QTLs in the Ciherang background, and was introduced into Sejong University (SJU, Seoul, Korea) by a Seconded Special Material Transfer Agreement (seconded SMTA), via Hankyong National University (HKNU, Anseong, Korea), with the IRRI (Los Baños, Philippines). IRBL9-w (Entry no. I22), derived from *O.minuta* (accession no. 101141), seed was obtained from Kyunghee University seed stock (KHU, Yongin, Korea). Initially, five CSA and three IRBL9-w plants were grown for crossing form which the cross between CSA-4 and IRBL9-w-2 was selected to develop CSA-Pi9. IR64-Sub1 + AG1 (GID 4537760) was introduced into SJU by seconded SMTA via HKNU with the IRRI. The IR64-Sub1 (Entry no. 961209) and IR64-AG1 (Entry no. 961212) genotypes, derived from IR64-Sub1 + AG1, were used as positive controls in the submergence and anaerobic germination screening experiments, respectively. IR64 (Entry no. 961061; accession no. IRGC 117268), obtained as a seed stock from SJU, was used as a susceptible control in the submergence and anaerobic germination screening experiments.

### Crossing Scheme for the Development of CSA-Pi9

CSA was crossed with IRBL9-w (Fig. 1), and 24 F_1_ plants were subjected to foreground genotyping to detect a functional allele of *Sub1*, *AG1* and *Pi9* using gene/QTL-specific markers: GnS2 for *Sub1* tolerant allele, DFR for *AG1* tolerant allele, ‘195’ for *Nbs2-Pi9* resistance allele and ‘NBS5’ for *Nbs5-Pi9* allele (Additional file 2: Table S1; Septiningsih et al. 2009; Kretzschmar et al. 2015; Qu et al. 2006). A single F_1_ plant showing the greatest phenotypic resemblance to CSA was backcrossed with CSA, and the resultant 94 BC_1_F_1_ plants were genotyped using foreground markers. Based on the genotyping results, a single plant was selected and backcrossed with CSA. Subsequently, 3 out of 288 BC_2_F_1_ plants were selected based on foreground genotyping results, and further characterized in a greenhouse for the heading date and phenotypic resemblance to CSA. Using the pedigree method, three BC_2_F_2_ breeding lines, morphologically resembling CSA and similar to CSA in agronomic traits including heading date, height and panicle, were selected, and the seeds of three plants of each line showing minimum segregation were propagated. BC_2_F_3_ plants were propagated in the field and advanced to the BC_2_F_5_ generation. Some of the BC_2_F_5_ seeds were used for the phenotypic screening of rice blast resistance, submergence tolerance and anaerobic germination. The BC_2_F_5_ plants were advanced further to the BC_2_F_7_ generation. The grain yield and quality of BC_2_F_5_ and BC_2_F_7_ plants were tested in the field in 2018 and 2019, respectively.

### Primer Design and Genotyping

All Nbs sequences of *Pi9* were compared, and two markers based on SNPs at nucleotide positions 659 and 1477 of *Nbs2-Pi9* were developed (Additional file 1: Fig. S1a). However, since the sequence of *Nbs5-Pi9* was identical to that of *Nbs2-Pi9* at the SNP position, as shown in the sequence alignment, only the Pi9_SNP1477G marker was used for MAS. The *Nbs2-Pi9*-specific primer (Qu et al. 2006; Additional file 2: Table S1) was used to distinguish between *Nbs2-Pi9* and *Nbs5-Pi9*.

Leaves were harvested from 2-week-old plants of CSA, CSA-Pi9, IR64, IR64-Sub1, and IR64-AG1 lines, and genomic DNA was extracted using the cetyltrimethylammonium bromide (CTAB) method (Murray and Thompson 1980). *Sub1*, *AG1*, and *Pi9* were genotyped by PCR using foreground markers (Additional file 2: Table S1). PCR was performed on the SimpliAmp thermocycler (Thermo Scientific, Waltham, MA, USA) in a 20-µL reaction volume, containing 50 ng of DNA template, 0.5 µL each of 10 µM forward and reverse primers (Bioneer, Daejeon, Korea), 2 µL of 10X buffer, 0.5 µL of 2.5 mM dNTPs and 0.1 µL of *Taq* polymerase (IN5001-0500; Inclone, Yongin, Korea), under the following conditions: initial denaturation at 94 °C for 4 min, followed by 30–35 cycles of denaturation at 94 °C for 1 min, annealing at 55–61 °C for 30–60 s, and extension at 72 °C for 30–60 s, with a final extension at 72 °C for 7 min. The PCR products were electrophoresed (BioFACT, Daejeon, Korea) on 1% or 2% agarose gel at 100–160 V for 20–40 min in 0.5X TBE buffer. Gels were visualized using the gel-imager (Korea Lab Tech, Seongnam, Korea).

Background genotyping of CSA-Pi9 and CSA was performed using the KNU Axiom Oryza 580 K Genotyping Array (Kim et al. 2019a, b) at DNAlink (Seoul, Korea). The KNU Axiom Oryza 580 K Genotyping Array consists of markers based on IRGSP v1.0 and MH63 v2. The results of background genotyping were analyzed based on the allele type at DNAcare (Seoul, Korea). The background recovery ratio was calculated as the number of markers with CSA alleles within 0.5 Mb segments of each chromosome relative to the total number of markers.

### Rice Blast Resistance Screening

CSA-Pi9 was challenged with *M. oryzae* isolates RO1-1 (compatible) and PO6-6 (incompatible). For blast resistance screening, the PO6-6 isolate was used to screen the plants of the early generations and both isolates (RO1-1 and PO6-6) were used to test the selected BC_2_F_5_-generation plants followed by the gene-specific markers of *Pi9*. Rice seedlings (F_1_ to BC_2_F_3_ generation) were planted individually in tubes. After 14–18 days of the growth, the seedlings were spray-inoculated with a spore suspension (1 × 10^6^ spores per mL) and incubated in a humid chamber for 14 days before assessing the resistance phenotype (Wu et al. 2015). The plants scored as highly resistant and moderately resistant were selected. The fully expanded leaves of 6-week-old plants (BC_2_F_5_- generation) were spot-inoculated with a spore suspension (2 × 10^6^ spores per mL), as described previously (Kanzaki et al. 2002), in 2018. The length of brown lesions on leaves was measured at 9 dpi.

### Submergence Tolerance and Anaerobic Germination Screening

Plants of CSA-Pi9, CSA (recurrent parent), IR64 (susceptible control), and IR64-Sub1 (resistant control) lines were grown for 2 weeks in a greenhouse, and submergence tolerance was tested as described previously (Septiningsih et al. 2009), with slight modifications. Briefly, seeds were surface-sterilized with a disinfectant in a 50-mL tube for 24 h, and then rinsed with water. The sterilized seeds were transferred to Petri dishes containing a moderate amount of water and incubated at 28 °C in the dark for 3 days. Pots (6 cm radius and 11 cm height) filled with air-dried soil were placed in a water-containing plastic box (20 cm height, 58.5 cm width, and 35 cm depth) for 1 day to soak the soil. Three seeds of each genotype were sown on the soil surface and covered with a 1-cm layer of soil. Forty-five seeds of each genotype were tested in a total of 15 pots, and 60 pots were arranged in a tray in a completely randomized design. Plants were grown under normal conditions for 2 weeks, with a constant water supply. The height and SPAD value of each seedling (total 45 seedlings) were measured at 14 days after transplanting (DAT). Then, 14-day-old plants were transferred to a 1-ton tank filled with 70-cm deep tap water. Water was drained from the tank after 14 days, and plants were covered with a fabric for 1 day to avoid dehydration under direct sunlight. Plants recovered for 7 days in a greenhouse under ambient conditions, and water was supplied to prevent drying. The height and SPAD value were measured at 14 days after submergence (DAS) using 21 seedlings of each genotype (The experiment started with 45 seedlings of each genotype. RNA sampling was performed at 6 points with 4 samples (3 samples of each genotype were used in experiment and 1 sample was extra against of sample loss), and remaining 21 seedlings of each genotype were evaluated.) Submergence screening was performed twice under natural conditions in 2018.

The germination of CSA-Pi9, CSA, IR64, and IR64-AG1 seeds was tested in 2018, as described previously (Angaji et al. 2010), with slight modifications. Two plastic boxes (20 cm height, 58.5 cm width, and 35 cm depth) were filled with an 8-cm layer of paddy field soil. Clods were broken apart with a trowel and sufficient volume of water. Each plastic box was divided into four sectors along its width using polyvinyl chloride (PVC) boards, and each sector was further divided into 10 sections along its length. To deoxidize the soil and water, one plastic box was placed in a chamber (anoxic conditions), while the other plastic box was placed in the greenhouse (hypoxic conditions) for 7 days. The chamber was maintained at a 33 °C day/28 °C night temperature under a 14-h light/10-h dark photoperiod, while the greenhouse was maintained under natural conditions. In each box, a total of 50 dried seeds of each genotype were sown in 10 rows (5 plants per row) at a soil depth of 1 cm. Water was added to each box every 3 days to maintain a 10-cm deep water layer throughout the experiment. The temperature of the water added to the box was approximately 30 °C to minimize oxygen provision. Seedlings that emerged above the water surface were counted, and the survival rate was calculated at 34 days after sowing. The coleoptile length and shoot length of three seedlings per genotype were measured at 3 days after the emergence of the last seedling. Anaerobic germination screening was conducted twice in the greenhouse and an environmentally controlled chamber, and genotypes were arranged in a completely randomized design in both experiments.

### RNA Extraction and Gene Expression Analysis

To analyze the expression *Sub1A*, *adh1*, and *OsTPP7* under submergence stress, three 14-day-old plants of each genotype (CSA-Pi9, CSA, IR64, and IR64-Sub1) were harvested at 0 (control; 14 days old plants), 1, 7, and 14 DAS. The harvested plants were rinsed with water in a tank, and excess water on the plants was soaked with a clean towel. The aerial and underground parts of each plant were separated and quickly frozen in liquid nitrogen. After 14 days, water was drained (de-submergence), and plant samples were collected at 1 and 7 DAR. To analyze the expression of *OsTPP7* and *adh1* during anaerobic germination, three seedlings of each genotype (CSA-Pi9, CSA, and IR64-AG1) were harvested at 34 days after sowing; IR64 seedlings could not be collected, as its seeds did not germinate.

Total RNA was extracted from the harvested aerial and underground parts of plants using the TRIzol Reagent (Thermo Fisher Scientific, MA, USA). First-strand cDNA (up to 1 µg/µL) was synthesized from the isolated total RNA using the Easy cDNA Synthesis Kit (NanoHelix, Daejeon, Korea), and diluted five times with distilled water. Quantitative PCR (qPCR) was performed on Mx3000 (Agilent, CA, USA) using the SensiFAST SYBR No-Rox Kit (Bioline, Meridian, London, UK). Each 20-µL qPCR reaction contained 200 ng of cDNA and 10 µM of sequence-specific forward and reverse primers (Additional file 2: Table S1; Bioneer, Daejeon, Korea); *Sub1A*-specific primers were designed in this study based on AAAA02037639.1, while primers used to amplify other genes have been published previously (Xu et al. 2006; Kretzschmar et al. 2015; Shin et al. 2020). The following conditions were used for qPCR: 95 °C for 10 min, followed by 40 cycles of 95 °C for 10 s, 61 °C for 15 s, and 72 °C for 20 s. Transcript levels of each gene were normalized to that of *OsUBQ5* using the 2^−ΔΔCt^ method (Livak and Schmittgen 2001).

### Field Evaluation of Plant Agronomic Traits

Plants of CSA-Pi9 and CSA genotypes were grown in field A (Suwon, Korea; 37° 16′ 08.7″ N 126° 59′ 24.0″ E) in 2018 and 2019, and in fields B and C (western coastal reclaimed regions in Seosan, Korea; field B: 36° 39′ 59.6″ N, 126° 26′ 16.0″ E; field C: 36° 40′ 01.0″ N, 126° 22′ 30.6″ E) in 2019. Plants in field A were grown under normal conditions in both years (2018 and 2019). Pre-germinated seeds of the two genotypes were sown in the greenhouse on April 27, 2018, and April 26, 2019, and only healthy 40-day-old seedlings were transplanted into the field at a spacing of 30 cm × 15 cm (row-to-plant). Fertilizer (N–P–K, 21–11–21 kg/1000m^2^) was applied on the day of transplanting, and conventional pesticide control was applied. In 2019, plants in field B were grown under normal irrigation conditions, while those in field C were grown under high salinity conditions. To generate plant materials for fields B and C, seeds were sown in the greenhouse on April 21, 2019. Then, 35-day-old seedlings were transplanted into fields B and C at a spacing of 30 cm × 15 cm (row-to-plant). Conventional practice was used to fertilize the plants in field B; however, plants in field C were treated with excess N fertilizer (N–P–K, 30–7–9 kg/1000 m^2^). Because of severe soil salinity, an active water circulation practice was applied to field C. The salinity, electrical conductivity (EC) and pH of water in field C were measured using a water quality meter (WM-32EP, TOADKK, Japan). Plants in all fields were grown in a completely randomized block design (6 rows × 30 plants per row). Two blocks, each consisting of 12 rows, were tested together in the whole plot.

Five plants of each genotype were selected for agronomic evaluation. Culm length (distance from the surface of the soil to the neck of the panicle) and panicle length (distance between the neck and tip of the longest panicle) were measured using the tallest tiller of each plant. The number of productive tillers per plant was counted to determine the panicle number. The average values of spikelet number, fertility, and hundred-grain weight were calculated from three panicles per plant. Fertility was calculated as the ratio of the fertile spikelet number to the total spikelet number. Grain yield per plant was calculated from the grain weight of three selected plants. In field C, the plants were harvested in bulk, thus grain yield was calculated by dividing total grain weight into the number of bulked plants.

Ten grains each of CSA-Pi9 and CSA were selected to examine grain traits. Grain length and grain width were measured using the Vernier caliper (Mitutoyo, Japan). Brown rice recovery was estimated by the ratio of the weight of dehulled rice to that of hulled grain. Chalkiness was scored using ImageJ (https://imagej.net/ImageJ). Area of the chalky region, which was shown in white color against the clear region, was estimated using the scanned image of brown rice of each genotype. Alkali spreading value was analyzed using 1.4% potassium hydroxide and scored as previously described (Kim and Kim 2016). Brown rice was ground using the CT 293 Cyclotec (FOSS, Hilleroed, Denmark), and amylose content was estimated using a Lambda 650 UV/VIS spectrometer (Perkin Elmer Inc., Walatham, USA) with a linear regression curve (R^2^ = 0.995) at an absorbance of 620 nm. The crude protein content of brown rice was estimated using DA 7250 NIR Analyzer (PerkinElmer, UK). The statistical analysis of data was conducted using R version 3.6.3 (R-Studio, MA, USA) and SPSS 16.0 (IBM, NY, USA).

## Supplementary Information

**Additional file 1: Fig. S1.** Identification of a unique single nucleotide polymorphism (SNP), *Nbs2-Pi9*, by multiple sequence alignment of nucleotide-binding sites (Nbss) of the *Pi9* gene cloned previously (Qu et al. 2006).

**Additional file 2: Table S1.** List of primers used in this study.

**Additional file 3: Table S2.** Statistics of CSA-Pi9-specific alleles on each chromosome.

**Additional file 4: Table S3.** List of SNPs possibly associated with salinity tolerance in CSA-Pi9.

## Data Availability

Datasets generated in the current study are available from the corresponding author upon reasonable request.

## Abbreviations

ABA: Abscisic acid
*adh1*: *Alcohol dehydrogenase 1*
Avr: Avirulence
CSA: Ciherang-Sub1 + AG1
CSA-Pi9: Ciherang-Sub1 + AG1 + Pi9
CTAB: Cetyltrimethylammonium bromide
DAR: Days after recovery
DAS: Days after submergence
DAT: Days after transplanting
dpi: Days post-inoculation
EC: Electrical conductivity
MABC: Marker-assisted backcrossing
MAS: Marker-assisted selection
NBS: Nucleotide-binding site
NBS-LRR: Nucleotide-binding site leucine-rich repeat
NIL: Near isogenic line
PVC: Polyvinyl chloride
qPCR: Quantitative PCR
QTL: Quantitative trait locus
SNP: Single nucleotide polymorphism
