# Supplementary material for: Development of a Temperate Climate-Adapted indica Multi-stress Tolerant Rice Variety by Pyramiding Quantitative Trait Loci
Source: Rice (N Y). 2022 Apr 9;15:22. doi: 10.1186/s12284-022-00568-2 (PMC8994804; doi:10.1186/s12284-022-00568-2)
Supplement: Supplementary file 1 — Additional file 1: Fig. S1. Identification of a unique single nucleotide polymorphism (SNP), Nbs2-Pi9, by multiple sequence alignment of nucleotide-binding sites (Nbss) of the Pi9 gene cloned previously (Qu et al. 2006). [file 12284_2022_568_MOESM1_ESM.pdf]

Development of a temperate climate-adapted *indica* multi-stress tolerant rice variety by pyramiding quantitative trait loci

Na-Hyun Shin[1,†], Jae-Hyuk Han[1,†], Kieu Thi Xuan Vo[2], Jeonghwan Seo[3], Ian Paul Navea[1,4], Soo-Cheul Yoo[5], Jong-Seong Jeon[2,*], Joong Hyoun Chin[1,*]

[*]Correspondence: jhchin@sejong.ac.kr; jjeon@khu.ac.kr

[1]Department of Integrative Biological Sciences and Industry, College of Life Sciences, Sejong University, Seoul, 05006, Korea

[2]Graduate School of Biotechnology and Crop Biotech Institute, Kyung Hee University, Yongin, Gyeonggi-do, 17104, Korea

[†]These authors contributed equally to this work.

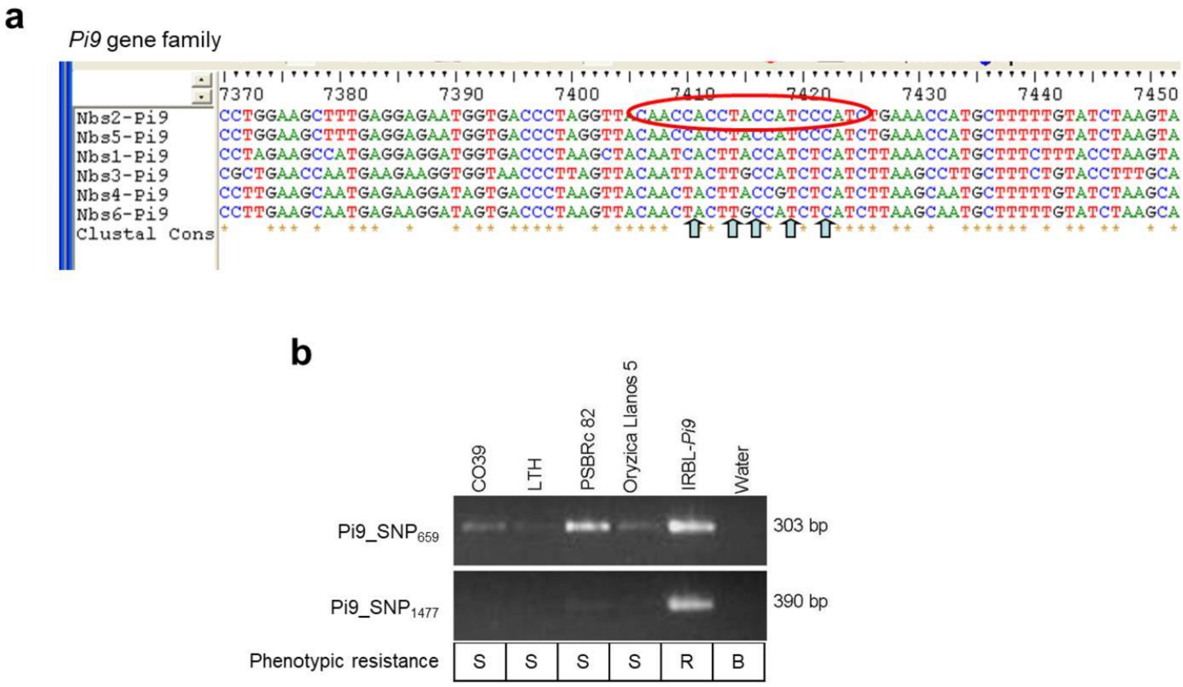

Supplementary Fig. S1 Identification of a unique single nucleotide polymorphism (SNP), *Nbs2-Pi9*, by multiple sequence alignment of nucleotide-binding sites (Nbss) of the *Pi9* gene cloned previously (Qu et al. 2006). Comparison of the *Pi9*-specific allele (*Nbs2-Pi9*) with other *Pi9* alleles for the development of the *Nbs2-Pi9*-specific marker (a). Validation of the developed markers using five rice varieties (b). Red circle indicates the *Nbs2-Pi9* allele. Arrows indicates the common single nucleotides among different Nbss.
